# Insurance Denials for Fluoride Varnish and Well-Child Visits

**DOI:** 10.1001/jamanetworkopen.2025.37086

**Published:** 2025-10-13

**Authors:** Ashley M. Kranz, Yuji Mizushima, Annie Yu-An Chen, Kun Li, Andrew W. Dick, Kimberley H. Geissler

**Affiliations:** 1 RAND, Arlington, Virginia; 2 RAND School of Public Policy, Santa Monica, California; 3 RAND, Boston, Massachusetts; 4 Duke-Margolis Institute for Health Policy, Duke University, Washington, DC; 5 University of Massachusetts Chan Medical School-Baystate, Springfield

## Abstract

This cross-sectional study examines rates of insurance claim denials for fluoride varnish treatment and well-child medical visits among US children.

## Introduction

The Patient Protection and Affordable Care Act (ACA) required insurers to cover evidence-based preventive services without patient cost-sharing. Despite this mandate, pediatricians report issues with reimbursement for fluoride varnish^1,2^—a treatment recommended by the US Preventive Services Task Force in 2014 for children 5 years and younger and reaffirmed in December 2021 for children 4 years and younger.^3,4^ Furthermore, claim denials are a problem more broadly.^5^ We used all-payer claims data (APCD) to examine claim denials for fluoride varnish applications and well-child medical visits (WCVs) for Medicaid and private insurers in Massachusetts.

## Methods

In this cross-sectional study, we analyzed 2022 medical claims from the Massachusetts APCD for fluoride varnish applications and WCVs from April to June 2025 (eMethods in Supplement 1). Private medical insurers are required to cover fluoride varnish through age 4 years, and Massachusetts’ Medicaid agency covers fluoride varnish through age 20 years. We examined fluoride varnish and WCV denials because fluoride varnish is frequently delivered during WCVs and WCVs are a common preventive service without cost-sharing.^6^ We used logistic regression models to estimate the probability of a denial, separately for fluoride varnish and WCV, controlling for insurance type and age group. We generated regression-adjusted probabilities and used average marginal effects to test differences by insurance type. Analyses were conducted using Stata/MP version 18.5 (StataCorp). This study followed the Strengthening the Reporting of Observational Studies in Epidemiology (STROBE) reporting guideline for cross-sectional studies and was approved by RAND’s Human Subjects Protections Committee with a waiver of informed consent.

## Results

Among 38 074 fluoride varnish claims and 776 741 WCV claims for children aged 11 years and younger, 33 895 fluoride varnish claims (89.0%) and 550 480 WCV claims (70.9%) were for children aged 5 years and younger, respectively. For ages 5 years and younger, fluoride varnish denial rates were low for Medicaid and private insurers (2% or below) (Figure, A), but private insurers were 0.80 percentage points (pp) (95% CI, 0.53-1.07 pp) more likely to deny fluoride varnish claims than Medicaid (Table)—corresponding to an 85% higher rate of denial compared with Medicaid. For ages 6 to 11 years, the denial rate remained low for Medicaid and increased to more than 20% for private insurers. Across all ages, WCV denial rates were more than 300% higher for private insurance than Medicaid (Figure, B).

**Figure.**
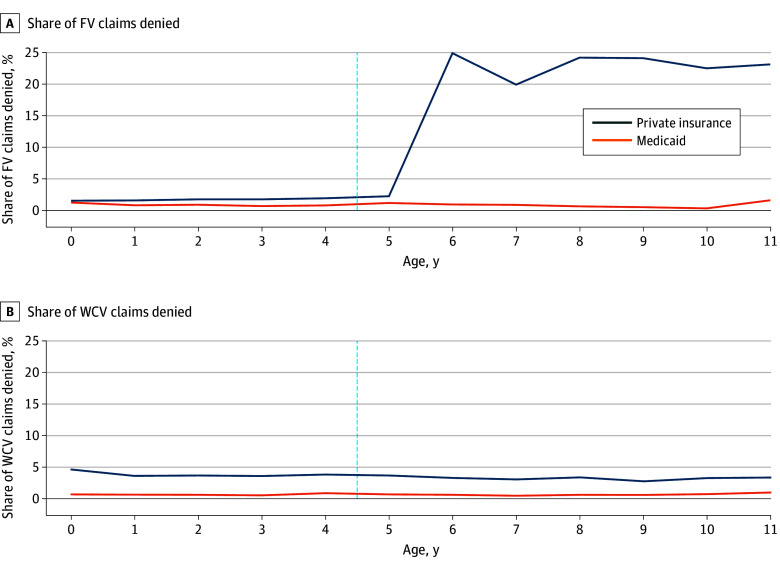
Total Claims and Share of Claims Denied for Fluoride Varnish (FV) and Well-Child Visits (WCVs), by Age and Insurance Type The vertical line between ages 4 and 5 years is included to emphasize that private plans were required to cover fluoride varnish without cost-sharing through age 5 years until November 2021 and through age 4 years starting December 2021.

**Table.** Projected Probabilities and Marginal Effects of Having a Claim Denied, Conditional on Insurance Type and Age Group^a^

Characteristics	Claim denials, %	
	Fluoride varnish	WCV
**Age 0-5 y**		
Private	1.74	4.21
Medicaid	0.94	0.74
Difference (marginal effect) (95% CI), percentage points	0.80 (0.53-1.07)^b^	3.47 (3.38-3.56)^b^
**Age 6-11 y**		
Private	23.79	3.26
Medicaid	0.88	0.74
Difference (marginal effect) (95% CI), percentage points	22.91 (19.30-26.52)^b^	2.52 (2.39-2.64)^b^

Abbreviation: WCV, well-child visit.

^a^ Adjusted projections were derived from logistic regression models accounting for insurance type, age group, and the interaction between these. Results for 95% CIs are heteroskedasticity robust.

^b^ *P* < .001.

## Discussion

In this study, private insurers were more likely than Medicaid to deny claims for children receiving fluoride varnish and WCVs, services that are mandated to be provided without patient cost-sharing.^6^ For patients, denials could result in unexpected costs, which is concerning because low-income and racial minority patients are more likely to have denied claims.^5^ For clinicians and practices, denials create administrative burdens, requiring time to determine if the claim should be corrected, appealed, or the patient billed. Policymakers should explore why the denial rates for these recommended services are higher among private insurers than Medicaid and whether insurers are passing along costs to consumers inappropriately.

Denials may be expected when a clinician bills insurance for a noncovered service. Private insurers are not required to cover fluoride varnish for children aged 5 years or older as of December 2021,^3^ but more than 75% of fluoride varnish claims for older children were not denied, suggesting that some private insurers continue to reimburse fluoride varnish. When plans cover different services or have different eligible populations, clinicians are unlikely to know what services are fully reimbursed, which could lead to unexpected costs for some patients and health care professionals. WCV denials are particularly concerning if they impact patient access to care.

Our cross-sectional study found that claim denials were higher for private insurers than Medicaid for common pediatric services and may contribute to increased patient costs and administrative burden. A study limitation is that insurers are not required to submit fully denied claims to the APCD. Our examination indicated most insurers submit multiple claims iterations, enabling us to identify denials. However, results may be a lower-end estimate of denials. Additionally, more research is needed to explore variation in findings across practice characteristics and insurers.

## Notes

Supplement 1. eMethods.

Supplement 2. Data Sharing Statement
