# Supplementary material for: Insurance Denials for Fluoride Varnish and Well-Child Visits
Source: JAMA Netw Open. 2025 Oct 13;8(10):e2537086. doi: 10.1001/jamanetworkopen.2025.37086 (PMC12519303; doi:10.1001/jamanetworkopen.2025.37086)
Supplement: Supplement 1. — eMethods. [file jamanetwopen-e2537086-s001.pdf]

Supplemental Online Content

Kranz AM, Mizushima Y, Chen AYA, Li K, Dick AW, Geissler KH. Insurance denials for fluoride varnish and well child visits. *JAMA Netw Open.* 2025;8(10):e2537086. doi:10.1001/jamanetworkopen.2025.37086

eMethods.

This supplemental material has been provided by the authors to give readers additional information about their work.

© 2025 Kranz AM et al. *JAMA Network Open*.

eMethods Supplement for "Insurance Denials for Fluoride Varnish and Well Child Visits"

Claims for fluoride varnish (FV) applications were identified using Current Procedural Terminology (CPT®) code 99188. Claims for well-child visits (WCVs) were identified using CPT® codes 99381-3 and 99391-3. We limited our sample to claims for services received in the state of Massachusetts and to claims that were not missing information on the insurance type or member ID. To ensure data quality, we examined the frequency of insurers reporting denied claims for FV and WCVs and dropped observations from two insurers reporting 0 denied claims and fewer than 21 claims total, respectively. This led to the dropping of 0.023% of claim lines for FV and 0.021% of claim lines for WCVs.

We examined FV denials and WCV denials because FV is frequently delivered during WCVs, WCVs are a common preventive service provided without patient cost-sharing due to the ACA and both are consistently observed in claims data. From prior work using the Massachusetts APCD, we know that more than 96% of FV applications occur during WCVs.[1] While other pediatric preventive services were required to be provided without patient cost-sharing due to the ACA (e.g., routine vaccines and a variety of screenings), the recommended periodicity of FV applications and WCVs are closely aligned, per guidance from Bright Futures/American Academy of Pediatrics,[2] and WCVs have procedure codes that are reliably reported and used for claims-based data analysis. A binary indicator of a denial was constructed with claims considered denied if the highest version claim line with the latest processing date was denied.

Children with private insurance are undercounted in the Massachusetts All-Payer Claims Database (APCD) compared to their true population in Massachusetts. This is because self-insured plans are not required to submit claims to the APCD starting in 2016 due to a Supreme

© 2025 Kranz AM et al. *JAMA Network Open*.

Court ruling.[3] In prior work using similar data, we found that approximately 60% of young children in these data have Medicaid,[1,4] which is higher than the estimated 35% of children aged 0-18 years in Massachusetts with Medicaid in 2022.[5]

References for eMethods

1. Chen AY-A, Geissler KH, Dick AW, Goff S, Kranz AM. Association between insurance type and fluoride varnish application during well-child visits in Massachusetts. *Academic pediatrics*. 2023;23(6):1213-1219.

2. Recommendations for Preventive Pediatric Health Care. Bright Futures/American Academy of Pediatrics. . August 5, 2025, https://downloads.aap.org/AAP/PDF/periodicity_schedule.pdf

3. Overview of the Massachusetts All-Payer Claims Database. Center for Health Information and Analysis. August 5, 2025, https://www.chiamass.gov/assets/docs/p/apcd/APCD-White-Paper-2016.pdf

4. Kranz AM, Chen AY-A, Mizushima Y, Li K, Dick AW, Geissler KH. Changes in Children's Receipt of Fluoride Varnish During Medical Visits and the COVID-19 Pandemic. *Academic Pediatrics*. 2025:102881.

5. State Health Facts: Health Insurance Coverage of Children 0-18. Kaiser Family Foundation. Accessed August 5, 2025, www.kff.org/other/state-indicator/children-0-18/?currentTimeframe=1&sortModel=%7B%22colId%22:%22Location%22,%22sort%22:%22asc%22%7D

© 2025 Kranz AM et al. *JAMA Network Open*.
