# Supplementary material for: Insurance Denials for Fluoride Varnish and Well-Child Visits
Source: JAMA Netw Open. 2025 Oct 13;8(10):e2537086. doi: 10.1001/jamanetworkopen.2025.37086 (PMC12519303; doi:10.1001/jamanetworkopen.2025.37086)
Supplement: Supplement 2. — Data Sharing Statement [file jamanetwopen-e2537086-s002.pdf]

Data Sharing Statement

Kranz. Insurance Denials for Fluoride Varnish and Well-Child Visits. *JAMA Netw Open*. Published October 13, 2025. doi:10.1001/jamanetworkopen.2025.37086

Data
Data available: No

Additional Information
Explanation for why data not available: We cannot share these data due to restrictions present in our data use agreement (DUA). These data can be obtained for research purposes through an application process with the Massachusetts Center for Health Information and Analysis.
